# Belgian validation of the INFLUENCE 3.0 model predicting 5-year locoregional and contralateral breast cancer recurrence

**DOI:** 10.1016/j.breast.2026.104944

**Published:** 2026-10-07

**Authors:** Aline van der Vorst, Carlijn Grimberg, Marissa C. van Maaren, Tom A. Hueting, Caroline Weltens, Sabine Siesling, Catharina G.M. Groothuis-Oudshoorn

**Affiliations:** a Department of Radiation Oncology, UZ Leuven, Herestraat 49, Leuven, 3000, Belgium; b Department of Health Technology and Services Research, Technical Medical Centre, University of Twente, Enschede, the Netherlands; c Department of Research and Development, Netherlands Comprehensive Cancer Organisation (IKNL), Utrecht, the Netherlands; d Evidencio Medical Decision Support, Haaksbergen, the Netherlands

**Keywords:** Breast cancer, Prediction, Recurrence, Contralateral breast cancer, External validation

## Abstract

**Background:**

Breast cancer (BC) surveillance primarily aims to detect locoregional recurrence (LRR) and contralateral breast cancer (CBC), but current strategies are largely one-size-fits-all and not tailored to individual risks. The Dutch INFLUENCE 3.0 prediction model provides personalised 5-year LRR and CBC risks to support shared decision-making on surveillance. In this study, INFLUENCE 3.0 is externally validated in a Belgian population.

**Methods:**

All female BC patients curatively treated between 2000 and 2018 at the University Hospitals of Leuven were included. Calibration was assessed using the Integrated Calibration Index (ICI), E50, E90 metrics, and calibration plots. Discrimination was evaluated by calculating the Area Under the Curve (AUC) for each year, based on cumulative events up to that year.

**Results:**

The final Leuven cohort included 8548 patients, of whom 7549 were not treated with neoadjuvant systemic therapy (non-NST) and 999 were (NST). Calibration metrics were generally acceptable, with ICI and E50 values below 0.01 in most subgroups. Calibration plots showed overestimation of predicted risks, particularly at lower predicted risk levels. Discrimination for LRR risk was good to very good (AUC 0.72–0.86 in the non-NST group; >0.82 in the NST group). For CBC risk, discrimination was poor in the non-NST group (AUC ≈ 0.5) and moderate-to-very good in the NST group (AUC 0.68–0.88).

**Conclusion:**

INFLUENCE 3.0 showed acceptable calibration and discrimination for LRR risk, and although CBC risk discrimination in the non-NST group was poor. The overall risk estimates – primarily the LRR risks – may still help in guiding tailored surveillance schemes.

## Highlights

• Breast cancer follow-up is currently not tailored to individual risks.
• INFLUENCE3.0 predicts 5-year locoregional and contralateral breast cancer recurrence.
• INFLUENCE supports decision-making on personalised breast cancer follow-up.
• INFLUENCE shows strong external validation for estimation of locoregional recurrence in Belgian data.
• Discrimination was poor for estimation of contralateral breast cancer.

## Graphical abstract

**Figure:**
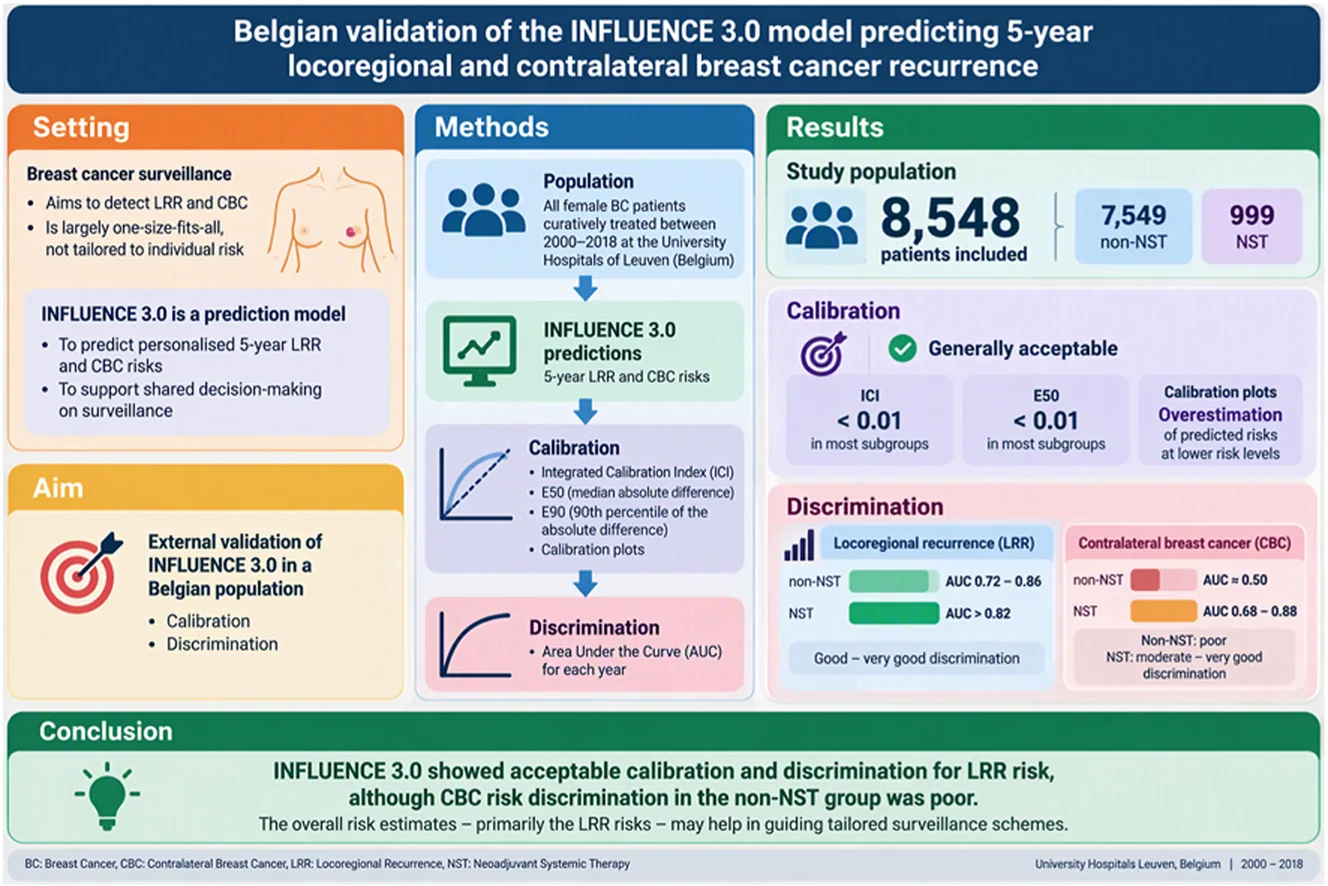

## 1 Introduction

In 2023, approximately 11,600 women in Belgium were diagnosed with invasive breast cancer (BC), most with early-stage disease [1]. Following curative treatment, patients enter surveillance primarily aimed at early detection of locoregional recurrence (LRR) and contralateral breast cancer (CBC). European guidelines recommend at least five years of follow-up with annual mammography and regular clinical visits [2]. Similarly, American guidelines recommend annual mammography and regular clinical visits for 3–10 years after treatment, with the duration of follow-up varying according to risk categories largely defined by tumor stage and subtype [3]. However, patients have substantial variation in recurrence risk related to additional factors such as age, tumor size, receptor subtype and treatment characteristics. With rising BC incidence and improved survival, this one-size-fits-all approach increases burden on healthcare and patient stress, while only a minority of recurrences are detected through scheduled surveillance [[4], [5], [6], [7], [8]]. A more risk-based personalised follow-up strategy to improve efficiency and reduce unnecessary burden for healthcare systems and patients is needed [[7], [8], [9]].

This demand led to the development of the Dutch prediction model INFLUENCE in 2015, subsequently updated to INFLUENCE 2.0 and most recently to INFLUENCE 3.0 [4,10,11]. INFLUENCE 3.0 provides individualised yearly risks of LRR and CBC within five years after surgery. In contrast to earlier versions, it is also applicable to patients treated with neoadjuvant systemic therapy (NST) and those with T4 disease. The model incorporates a wide range of clinicopathologic and treatment-related variables including age, mode of detection, histology, sublocalisation, grade, pT, pN, hormonal receptor status ± endocrine treatment, HER2 status ± targeted treatment, surgery ± immediate reconstruction ± radiation therapy, chemotherapy, presence of ductal carcinoma in situ, axillary lymph node dissection and pathologic complete response (in case of NST). It is available as a CE-certified online tool via the Evidencio platform [12]. INFLUENCE supports shared decision-making on personalised surveillance and is currently used in clinical practice within the multicenter NABOR study evaluating the (cost-)effectiveness of personalised follow-up [13]. The INFLUENCE 3.0 model was previously internally validated on the Dutch BC population and demonstrated adequate predictive performance [8].

Before broader clinical implementation, INFLUENCE 3.0 requires external validation in independent populations [[14], [15], [16], [17]]. Therefore, the objective of this study is to externally validate INFLUENCE 3.0 on patient data from the University Hospitals Leuven, Belgium. The results provide insight into the robustness of INFLUENCE 3.0 and thus will indicate whether the model could be suitable for broader use across different populations and healthcare settings.

## 2 Methods

### 2.1 Ethical considerations

This study was approved by the Ethics Committees of the University of Twente (240722) and the University Hospitals Leuven (S68978), with a non-commercial Data Transfer Agreement in place.

### 2.2 Data acquisition

Data were obtained from the University Hospitals Leuven, a tertiary referral centre in Belgium, where clinical and treatment data, including follow-up information, are prospectively collected through the multidisciplinary breast cancer centre. Patients with breast cancer treated at the University Hospitals Leuven continue their follow-up at our institution for up to 10 years after treatment. During the first five years, follow-up consists of biannual clinical evaluations, annual mammography, and CA 15-3 (tumor marker) testing. During the subsequent five years, clinical examinations are performed every nine months, while mammography and CA 15-3 testing are performed every 18 months. This comprehensive follow-up protocol resulted in extensive treatment and follow-up data with limited missingness. Female patients with unilateral BC treated with curative intent between 2000 and 2018 were included, yielding 8742 patients. Data were pseudonymized and securely transferred to the University of Twente. Exclusion criteria from INFLUENCE 3.0 development were applied (synchronous BC, positive surgical margins, unknown surgery date or incomplete follow-up).

### 2.3 Outcomes and definitions

“The primary outcomes were LRR and CBC, which were defined as reappearance of breast cancer on the ipsilateral side or regional nodes (LRR) or the contralateral breast (CBC) [18].” Individual follow-up was calculated from definitive surgery to the first event, distant metastasis, death, or last observation. Events occurring within 30 days were counted jointly; otherwise, only the first event was considered. Patients were censored at the time another event occurred (distant metastasis or death), with these events treated as competing events [19], and follow-up was truncated at five years.

Missing data were assumed missing at random and imputed five times using the *mice* package in R (Appendix A) [20].

### 2.4 Validation

For each imputed dataset, quarterly individual LRR and CBC risks were estimated using the appropriate INFLUENCE 3.0 submodel. Calibration and discrimination of the four submodels (LRR and CBC, analyzed separately for patients with and without NST (hereafter referred to as NST and non-NST) were evaluated and compared with internal validation results.

Calibration was assessed using the Integrated Calibration Index (ICI) (weighted difference between predicted and observed probabilities), E50 (median absolute difference), and E90 (90th percentile of the absolute difference), calculated annually and compared against internal validation values [10]. Lower values indicate better calibration, with ICI <0.01 considered good. Calibration was assessed using a Cox model with three restricted cubic splines and complementary log–log–transformed predicted risks, following Austin et al. [21].

In addition, calibration plots were generated at five years using smoothed curves with bootstrap CI's and decile-based Kaplan–Meier estimates [17,22]. Discrimination was assessed annually using time-dependent area under the curves (AUCs) at 1, 2, 3, 4 and 5 years from date of definitive surgery, and were classified as poor (0.5–0.6), moderate (0.6-0.7), good (0.7–0.8), or very good (≥0.8), with corresponding CIs [23].

Results from the five imputed datasets were pooled using Rubin's Rules [24,25].

## 3 Results

### 3.1 Patient characteristics

After the exclusion of several patients (Fig. 1), the final cohort consisted of 8548 patients: 7549 who did not receive neoadjuvant systemic therapy (non-NST group) and 999 who did (NST group). Baseline and treatment-related characteristics for both groups are summarized in Table 1.

**Fig. 1:**
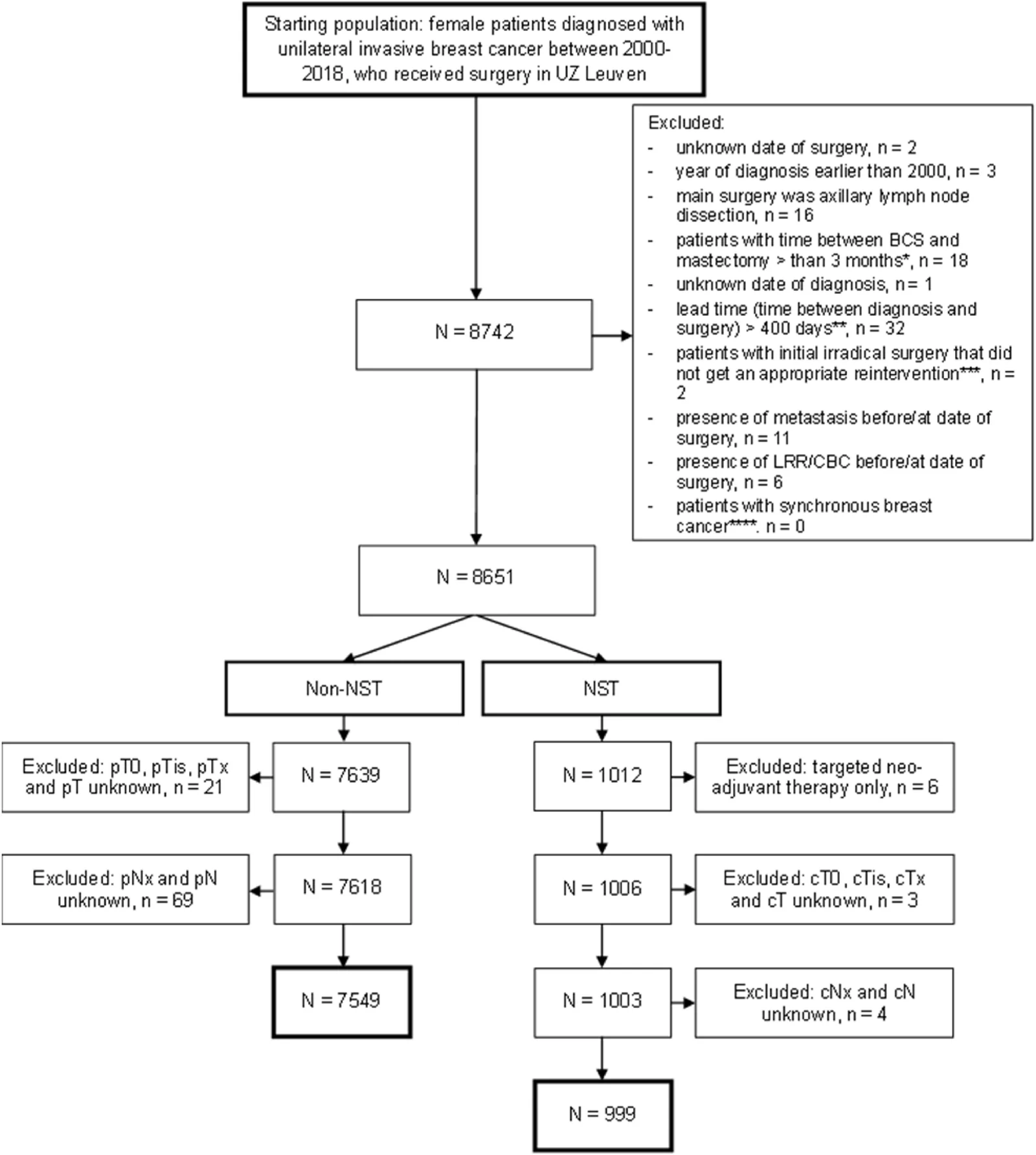
Flowchart of patient selection and exclusion * Decided in consultation with a clinician. Among these 18 cases, most had intervals much longer than 3 months (e.g. years) between BCS and mastectomy, suggesting prior breast cancer. Also, irradical margins may cause mastectomy after BCS is performed, but this should occur shortly after the initial BCS. ** Decided in consultation with a clinician. A lead time exceeding 400 days was considered implausible for both non-NST and NST patients. *** Initially, approximately 300 patients had irradical surgery. In the INFLUENCE 3.0 development, patients with focally irradical margins and those receiving re-intervention resulting in clean margins were included. A random sample of 50 patients was reviewed; only 2 had more than focal irradical margins without appropriate re-intervention. Based on this, and in agreement with a clinician, the remaining 250 patients were assumed to have either focal irradicality or adequate re-intervention. **** Second primary breast tumour diagnosed within 90 days of the first. Abbreviations: LRR = locoregional recurrence, CBC = contralateral breast cancer, NST = neoadjuvant systemic treatment, BCS = breast conserving surgery, p = pathological (post-operative), c = clinical (pre-treatment), T = tumour, N = nodal, is = in situ, x = unknown/could not be assessed.

**Table 1:** Overview of patient, tumour and treatment characteristics in the Leuven cohort divided into two groups: patients treated without neoadjuvant systemic treatment (non-NST) and patients treated with neoadjuvant systemic treatment (NST).

	No neoadjuvant systemic treatment (non-NST) (n = 7549)	Neoadjuvant systemic treatment (NST) (n = 999)
**Year of diagnosis**		
2000/2001	730 (9,7%)	51 (5,1%)
2002/2003	802 (10,6%)	60 (6,0%)
2004/2005	840 (11,1%)	68 (6,8%)
2006/2007	879 (11,6%)	81 (8,1%)
2008/2009	879 (11,6%)	99 (9,9%)
2010/2011	779 (10,3%)	105 (10,5%)
2012/2013	824 (10,9%)	127 (12,7%)
2014/2015	718 (9,5%)	169 (16,9%)
2016/2017	735 (9,7%)	159 (15,9%)
2018	363 (4,8%)	80 (8,0%)
**Age (years)**		
median (IQR)	58 (50 – 68)	53 (44 - 64)
< 40	411 (5,4%)	160 (16,0%)
40 – 49	1434 (19,0%)	245 (24,5%)
50 – 59	2151 (28,5%)	260 (26,0%)
60 – 69	1974 (26,1%)	167 (16,7%)
70 – 79	1108 (14,7%)	128 (12,8%)
> 79	471 (6,2%)	39 (3,9%)
**Mode of detection**		
clinically detected	4465 (59,1%)	834 (83,5%)
screen-detected	2763 (36,6%)	91 (9,1%)
unknown	321 (4,3%)	74 (7,4%)
**Sublocalisation**		
outer quadrants	3425 (45,4%)	448 (44,8%)
inner quadrants	1527 (20,2%)	146 (14,6%)
central parts	213 (2,8%)	42 (4,2%)
overlapping lesions	1959 (26,0%)	244 (24,4%)
unknown	425 (5,6%)	119 (11,9%)
**Histological tumour type**		
ductal	5898 (78,1%)	875 (87,6%)
lobular	995 (13,2%)	72 (7,2%)
mixed ductal lobular	87 (1,2%)	9 (0,9%)
other	569 (7,5%)	43 (4,3%)
**Differentiation grade**		
grade 1	1295 (17,2%)	42 (4,2%)
grade 2	3550 (47,0%)	411 (41,1%)
grade 3/4	2659 (35,2%)	526 (52,7%)
unknown	45 (0,6%)	20 (2,0%)
**Clinical tumour stage**		
cTIS	100 (1,3%)	-
cT0	20 (0,3%)	-
cT1	3599 (47,7%)	95 (9,5%)
cT2	3214 (42,6%)	363 (36,3%)
cT3	429 (5,7%)	209 (20,9%)
cT4	72 (1,0%)	332 (33,2%)
unknown	115 (1,5%)	-
**Clinical nodal stage**		
cN0	6245 (82,7%)	296 (29,6%)
cN1	1176 (15,6%)	399 (39,9%)
pN2	26 (0,3%)	97 (9,7%)
pN3	15 (0,2%)	207 (20,7%)
unknown	87 (1,2%)	-
**Pathological tumour stage (ypT in case of NST)**		
pT0	-	219 (21,9%)
pTIS	-	95 (9.5%)
pT1	3815 (50,5%)	238 (23,8%)
pT2	3141 (41,6%)	244 (24,4%)
pT3	545 (7,2%)	166 (16,6%)
pT4	48 (0,6%)	22 (2,2%)
unknown	-	15 (1,5%)
**Pathological nodal stage**		
pN0	4758 (63,0%)	481 (48,1%)
pN1	2116 (28,0%)	244 (24,4%)
pN2	462 (6,1%)	122 (12,2%)
pN3	213 (2,8%)	97 (9,7%)
unknown	-	55 (5,5%)
**Hormonal receptor status + treatment**		
positive + endocrine therapy	6302 (83,5%)	562 (56,3%)
positive – endocrine therapy	252 (3,3%)	17 (1,7%)
negative	976 (12,9%)	415 (41,5%)
unknown	19 (0,3%)	5 (0,5%)
**HER2 status + treatment**		
negative	6585 (87,2%)	683 (68,4%)
positive + targeted therapy	549 (7,3%)	269 (26,9%)
positive – targeted therapy	314 (4,2%)	38 (3,8%)
unknown	101 (1,3%)	9 (0,9%)
**Ductal carcinoma in situ present**		
no	2037 (27,0%)	493 (49,3%)
yes	5512 (73,0%)	506 (50,7%)
**Axillary lymph node dissection**		
no	3250 (43,1%)	163 (16,3%)
yes	4299 (56,9%)	836 (83,7%)
**Type of surgery + RT + immediate breast reconstruction**		
BCS + RT	4049 (53,6%)	234 (23,4%)
BCS – RT mastectomy + RT + direct reconstruction	57 (0,8%)	5 (0,5%)
mastectomy + RT + direct reconstruction	2254 (29,9%)	691 (69,2%)
mastectomy – RT – direct reconstruction	80 (1,1%)	11 (1,1%)
mastectomy – RT + direct reconstruction	1008 (13,4%)	55 (5,5%)
	101 (1,3%)	3 (0,3%)
**Chemotherapy**		
no	4768 (63,2%)	94 (9,4%)
yes	2781 (36,8%)	905 (90,6%)
**Pathologic complete response**		
no	NA	720 (72,1%)
yes	NA	275 (27,5%)
unknown	NA	4 (0,4%)
**Vital status at end of follow-up**		
alive	5556 (73,3%)	673 (67,4%)
deceased (all causes)	1993 (26,4%)	326 (32,6%)
**Locoregional recurrence as first event**^a^		
no	7324 (97%)	960 (96,1%)
yes	225 (3%)	39 (3,9%)
**Contralateral breast tumour as first event**^a^		
no	7234 (95,8%)	971 (97,2%)
yes	315 (4,2%)	28 (2,8%)

Abbreviations: NST = neoadjuvant systemic treatment, IQR = interquartile range, c = clinical (pre-operative), p = pathological (post-operative), T = tumour, N = nodal, IS = in situ, HER2 = human epidermal growth factor receptor 2, NA = not applicable, BCS = breast-conserving surgery, RT = radiation therapy.

a In the first 5 years after surgery, there were 93 and 28 LRR cases in the non-NST and NST group, and 86 and 13 CBC cases respectively.

Within five years, LRR occurred in 121 (1.4%) and CBC in 99 (1.2%) patients, with slightly higher LRR and CBC rates in the NST group (1.2% non-NST versus 2.8% NST and 1.1% non-NST versus 1.3% NST, respectively).

### 3.2 Calibration

Yearly ICI, E50, and E90 values by subgroup, which generally increased over time (Table 2). All ICI and E50 values were <0.01 except for LRR in the NST group; E90 values were naturally higher but remained close to zero. Calibration was slightly less favorable than in internal validation [10].

**Table 2:** Overview of the ICI, E50 and E90 for every year, for each subgroup.

		Year	LB	ICI	UB	LB	E50	UB	LB	E90	UB
**LRR**	**non-NST**	1	0.0001	**0.0005**	0.0013	0.0000	**0.0003**	0.0007	0.0000	**0.0004**	0.0021
		2	0.0005	**0.0013**	0.0025	0.0002	**0.0009**	0.0018	0.0002	**0.0020**	0.0047
		3	0.0007	**0.0018**	0.0036	0.0003	**0.0013**	0.0025	0.0004	**0.0024**	0.0058
		4	0.0010	**0.0025**	0.0047	0.0006	**0.0019**	0.0035	0.0008	**0.0040**	0.0082
		5	0.0012	**0.0030**	0.0056	0.0007	**0.0022**	0.0042	0.0011	**0.0048**	0.0096
	**NST**	1	0.0018	**0.0045**	0.0110	0.0017	**0.0047**	0.0085	0.0000	**0.0055**	0.0264
		2	0.0032	**0.0105**	0.0201	0.0034	**0.0073**	0.0127	0.0000	**0.0248**	0.0606
		3	0.0049	**0.0135**	0.0244	0.0050	**0.0101**	0.0164	0.0000	**0.0277**	0.0639
		4	0.0075	**0.0177**	0.0298	0.0078	**0.0139**	0.0212	0.0000	**0.0316**	0.0688
		5	0.0091	**0.0194**	0.0316	0.0099	**0.0164**	0.0239	0.0016	**0.0322**	0.0692
**CBC**	**non-NST**	1	0.0005	**0.0012**	0.0019	0.0003	**0.0011**	0.0018	0.0013	**0.0021**	0.0028
		2	0.0004	**0.0011**	0.0023	0.0001	**0.0007**	0.0021	0.0009	**0.0029**	0.0051
		3	0.0013	**0.0027**	0.0045	0.0008	**0.0029**	0.0049	0.0022	**0.0049**	0.0079
		4	0.0021	**0.0040**	0.0063	0.0018	**0.0045**	0.0069	0.0029	**0.0060**	0.0099
		5	0.0031	**0.0052**	0.0079	0.0025	**0.0058**	0.0087	0.0042	**0.0083**	0.0132
	**NST**	1	0.0000	**0.0015**	0.0064	0.0000	**0.0013**	0.0040	0.0000	**0.0029**	0.0145
		2	0.0011	**0.0030**	0.0083	0.0017	**0.0024**	0.0069	0.0000	**0.0049**	0.0161
		3	0.0021	**0.0058**	0.0117	0.0031	**0.0061**	0.0089	0.0000	**0.0083**	0.0241
		4	0.0031	**0.0047**	0.0104	0.0029	**0.0037**	0.0104	0.0044	**0.0088**	0.0168
		5	0.0030	**0.0079**	0.0161	0.0038	**0.0088**	0.0135	0.0015	**0.0110**	0.0305
		Year	LB	**ICI**	UB	LB	**E50**	UB	LB	**E90**	UB

**LRR**	**non-NST**	1	0.0001	**0.0005**	0.0013	0.0000	**0.0003**	0.0007	0.0000	**0.0004**	0.0021
		2	0.0005	**0.0013**	0.0025	0.0002	**0.0009**	0.0018	0.0002	**0.0020**	0.0047
		3	0.0007	**0.0018**	0.0036	0.0003	**0.0013**	0.0025	0.0004	**0.0024**	0.0058
		4	0.0010	**0.0025**	0.0047	0.0006	**0.0019**	0.0035	0.0008	**0.0040**	0.0082
		5	0.0012	**0.0030**	0.0056	0.0007	**0.0022**	0.0042	0.0011	**0.0048**	0.0096
	**NST**	1	0.0018	**0.0045**	0.0110	0.0017	**0.0047**	0.0085	0.0000	**0.0055**	0.0264
		2	0.0032	**0.0105**	0.0201	0.0034	**0.0073**	0.0127	0.0000	**0.0248**	0.0606
		3	0.0049	**0.0135**	0.0244	0.0050	**0.0101**	0.0164	0.0000	**0.0277**	0.0639
		4	0.0075	**0.0177**	0.0298	0.0078	**0.0139**	0.0212	0.0000	**0.0316**	0.0688
		5	0.0091	**0.0194**	0.0316	0.0099	**0.0164**	0.0239	0.0016	**0.0322**	0.0692
**CBC**	**non-NST**	1	0.0005	**0.0012**	0.0019	0.0003	**0.0011**	0.0018	0.0013	**0.0021**	0.0028
		2	0.0004	**0.0011**	0.0023	0.0001	**0.0007**	0.0021	0.0009	**0.0029**	0.0051
		3	0.0013	**0.0027**	0.0045	0.0008	**0.0029**	0.0049	0.0022	**0.0049**	0.0079
		4	0.0021	**0.0040**	0.0063	0.0018	**0.0045**	0.0069	0.0029	**0.0060**	0.0099
		5	0.0031	**0.0052**	0.0079	0.0025	**0.0058**	0.0087	0.0042	**0.0083**	0.0132
	**NST**	1	0.0000	**0.0015**	0.0064	0.0000	**0.0013**	0.0040	0.0000	**0.0029**	0.0145
		2	0.0011	**0.0030**	0.0083	0.0017	**0.0024**	0.0069	0.0000	**0.0049**	0.0161
		3	0.0021	**0.0058**	0.0117	0.0031	**0.0061**	0.0089	0.0000	**0.0083**	0.0241
		4	0.0031	**0.0047**	0.0104	0.0029	**0.0037**	0.0104	0.0044	**0.0088**	0.0168
		5	0.0030	**0.0079**	0.0161	0.0038	**0.0088**	0.0135	0.0015	**0.0110**	0.0305

Abbreviations: E50 = median absolute difference. E90 = 90th percentile of the absolute difference. ICI = Integrated Calibration Index. LB = 95% confidence interval lower bound. UB = 95% confidence interval upper bound.

Predicted LRR risks were generally low. Median LRR risk was 0.95% (IQR 0.54–1.70%) in non-NST and 3.08% (IQR 2.57–4.71%) in NST patients. For the majority of predictions up to ∼5%, calibration curves lay below the diagonal, with CIs widening at higher risks, especially in the NST group (Fig. 2).

**Fig. 2:**
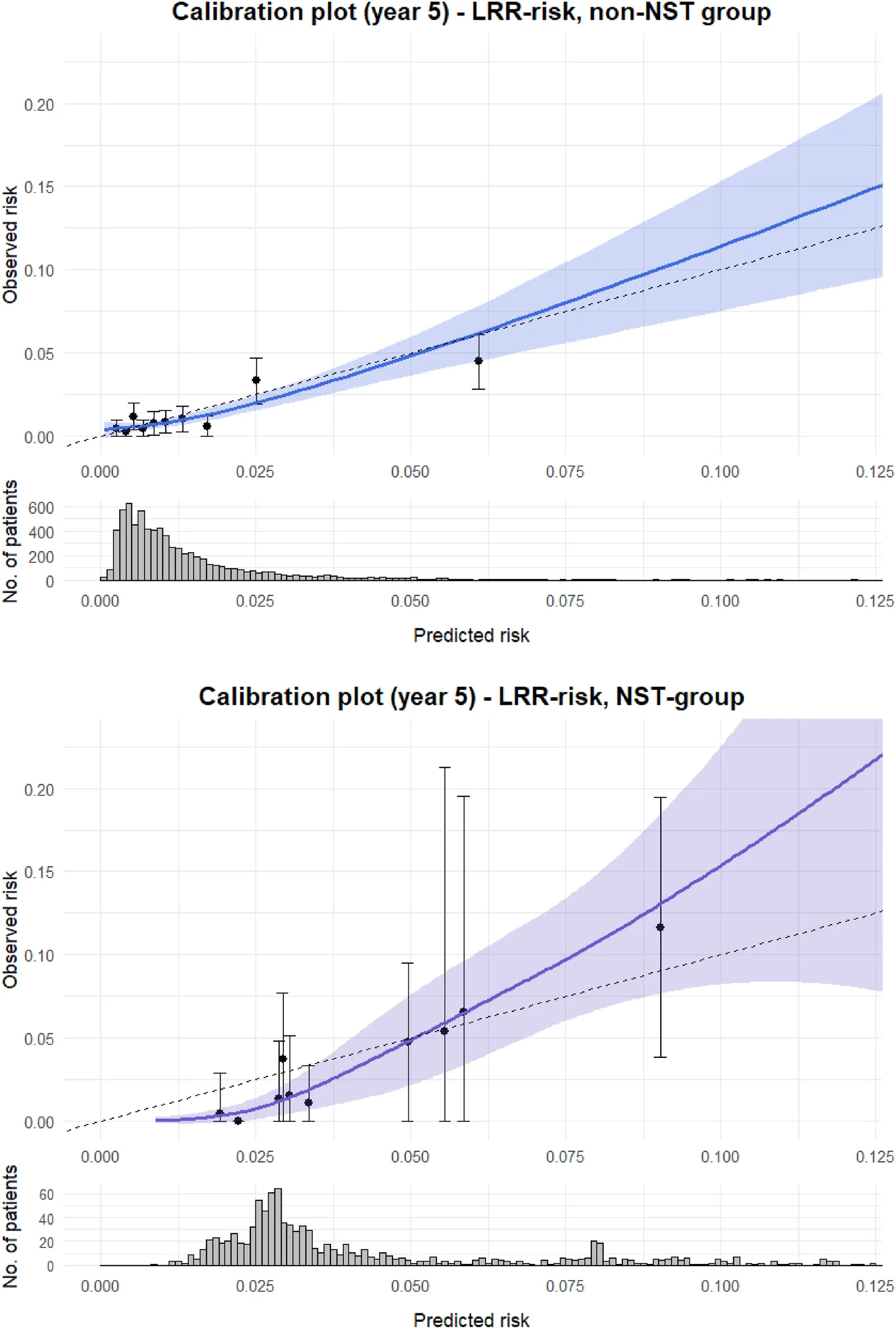
Calibration plots for 5-year LRR risk estimation (non-NST group and NST group).

Most predicted CBC risks were 1–2% (Fig. 3). Median risk was 1.70% (IQR 1.43–1.90%) in non-NST and 1.60% (IQR 1.23–1.95%) in NST patients. In non-NST, the calibration curve stayed below the diagonal, while in NST it rose above 1.5–2.3% before falling again. CIs were wider in the NST group.

**Fig. 3:**
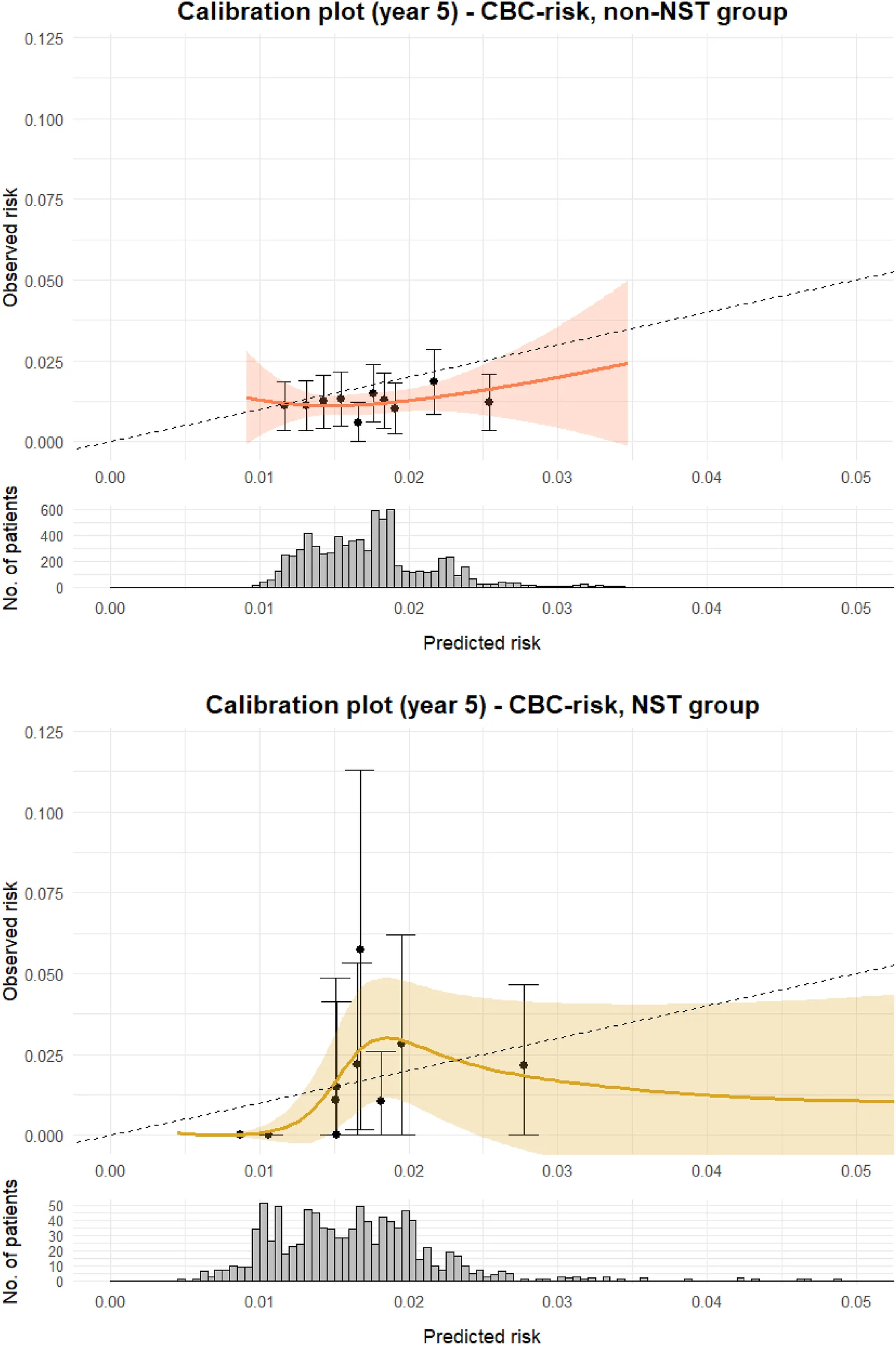
Calibration plots for 5-year CBC risk estimation (non-NST group and NST group).

### 3.3 Discrimination

Overall, AUCs declined slightly over time (Fig. 4). For LRR, AUCs were >0.8 in years 1–2 and 0.7–0.8 in years 3–5 in the non-NST group. In NST patients, AUCs remained >0.8 each year, exceeding internal validation values (Table 3).

**Fig. 4:**
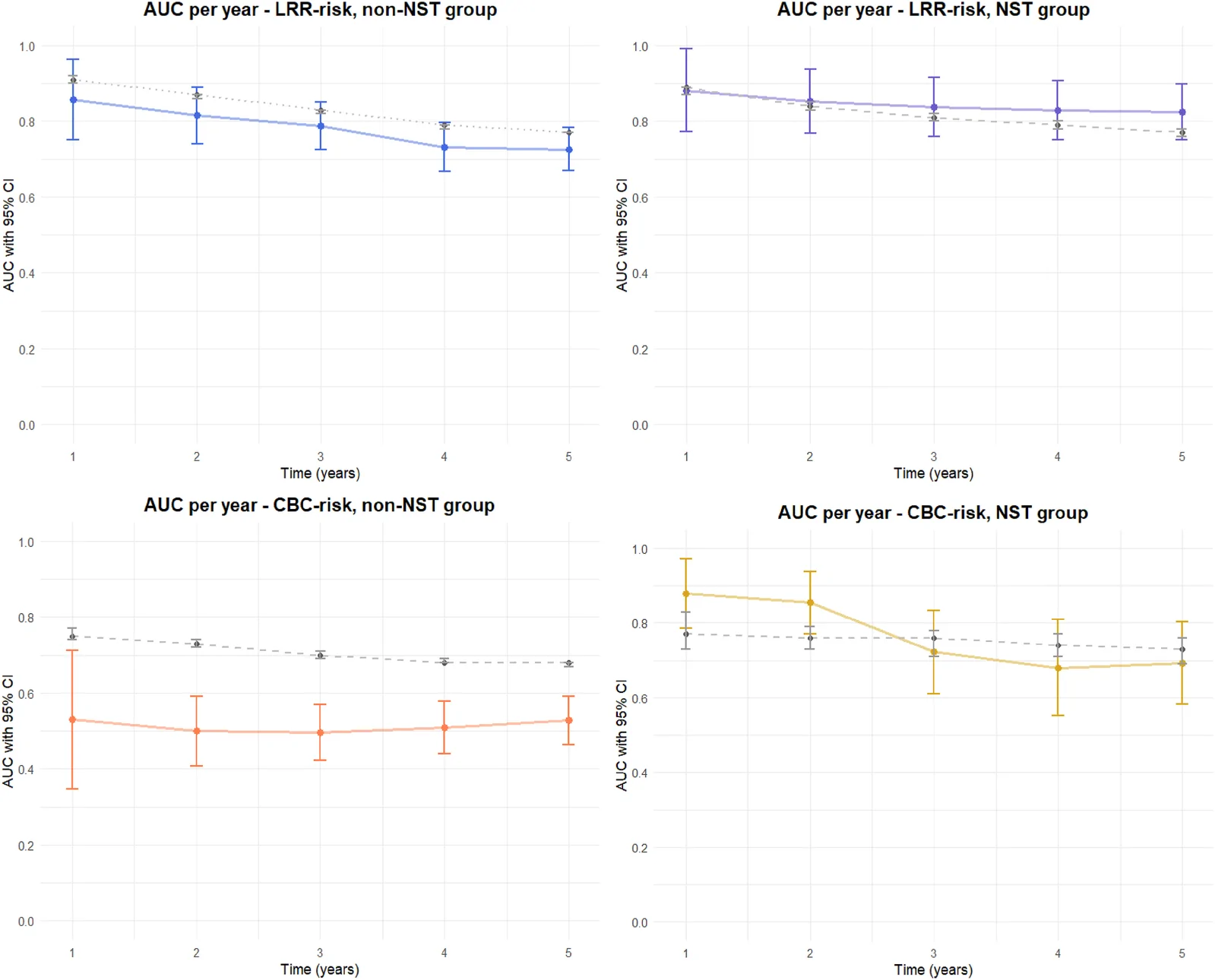
Yearly AUCs for every subgroup. In grey the internal validation results of INFLUENCE 3.0 [6] are shown.

**Table 3:** Discrimination results per year.

		Time (years)	LB	AUC	UB
**LRR**	**non-NST**	1	0.751	**0.857**	0.963
		2	0.741	**0.816**	0.890
		3	0.724	**0.788**	0.851
		4	0.667	**0.731**	0.796
		5	0.668	**0.726**	0.784
	**NST**	1	0.772	**0.881**	0.990
		2	0.769	**0.853**	0.938
		3	0.760	**0.838**	0.915
		4	0.751	**0.829**	0.906
		5	0.751	**0.825**	0.899
**CBC**	**non-NST**	1	0.347	**0.529**	0.711
		2	0.407	**0.499**	0.591
		3	0.422	**0.496**	0.570
		4	0.439	**0.508**	0.578
		5	0.464	**0.528**	0.592
	**NST**	1	0.786	**0.879**	0.972
		2	0.771	**0.854**	0.937
		3	0.611	**0.722**	0.833
		4	0.552	**0.680**	0.808
		5	0.582	**0.692**	0.802

Abbreviations: LB = 95% confidence interval lower bound. AUC = (mean) area under the receiver operating characteristic curve. UB = 95% confidence interval upper bound.

For CBC, non-NST AUCs remained around 0.5 over five years, lower than internal validation. In NST, AUCs were >0.8 in years 1–2 and ∼0.7 thereafter.

## 4 Discussion

### 4.1 Evaluation of the results

Calibration of the four INFLUENCE 3.0 submodels was generally good, with the ICI and E50 below 0.01 for all models except the LRR risk estimation in the NST group, where the ICI slightly exceeded this threshold, indicating somewhat reduced calibration. However, ICI, E50, and E90 are best interpreted comparatively, as no universally established reference values exist [21]. The threshold of 0.01 was therefore used as a point of reference, in line with the internal validation of INFLUENCE 3.0 [8]. Moreover, calibration measures depend on the distribution of predicted risks. As the LRR submodel in the NST group generated higher predicted risks, similar degrees of miscalibration may result in larger absolute differences, leading to higher ICI, E50, and E90 values.

For the LRR model in the NST group, calibration plots indicated general overestimation of predicted risks compared to observed outcomes. The bootstrap-based CI did not include the diagonal in the low predicted risk range, suggesting significant miscalibration in this region. As bootstrap-based calibration curves avoid information loss due to risk grouping and are considered more robust, they were used as the primary representation of calibration performance [17,21]. The complementary decile-based plot showed wider CIs, reflecting greater uncertainty, likely due to the small number of events (n = 28) [17,26]. For the CBC risk estimation in the NST group, the calibration curve was irregular and non-monotonic, diverging from the diagonal across different regions, again likely driven by the very small number of events (n = 13).

In the non-NST group, calibration metrics for the CBC model were good, although the calibration curve consistently underestimated observed risks, with the CI falling below the diagonal over a substantial range. However, absolute deviations from perfect calibration were small, remaining below 0.01 across the entire risk range. Calibration of the Cox proportional hazards model for LRR risk estimation in the non-NST group was best. This may be partly explained by Cox models being generally less prone to overfitting and yielding more stable risk estimates than random survival forest models [8,27,28].

For external validation studies, some degree of miscalibration is expected [14,17,29]. In this study, differences in case-mix and incidence rates likely contributed to the observed miscalibration [14,17]. Specifically, the generally lower five-year event rates in the Leuven cohort (1.4% LRR and 1.2% CBC) compared to the Dutch development population (2.2% LRR and 1.4% CBC) may explain part of the risk overestimation [14,[29], [30], [31], [32]].

Discriminative performance was the most important aspect of the model evaluation. Overall, the AUCs for LRR were good in both groups (range 0.73–0.88). As expected, AUCs decreased over time, consistent with internal validation results and the declining predictive ability further from baseline [33].

CBC risk estimation showed markedly poorer performance, particularly in the non-NST group, with AUCs around 0.5, indicating discrimination no better than chance [17]. In the NST group, CBC discrimination was good at three years but declined to moderate performance at four and five years.

The reasons for this weak performance are difficult to pinpoint. Case-mix variation in the external cohort appeared adequate, but CBC risk prediction is inherently challenging due to the limited predictive power of established risk factors and the influence of lifestyle variables [34]. Other known CBC predictors—germline mutations, family history, BMI, and young-age RT—are not included in INFLUENCE 3.0 [35]. However, even models incorporating some of these factors, such as PredictCBC-2.0, achieved only moderate AUCs (∼0.65) in large cohorts [34]. A sensitivity analysis, in a more homogeneous and less historical cohort, did not improve performance, suggesting that changes in treatment policy do not explain the findings (Appendix B).

The original INFLUENCE 1.0 model, which predicted five-year LRR in non-NST–treated patients only, was externally validated in a German cohort. Of course, a direct comparison with our validation is not possible because the updated INFLUENCE 3.0 model additionally predicts for CBC, is applicable to both NST and non-NST patients and used different methodology. Nevertheless, the German validation showed that the original model had good total discrimination (AUC 0.73; 95% CI 0.69–0.77), but performance declined annually, reaching no meaningful discrimination by 5 years (AUC 0.50; 95% CI 0.41–0.60). Calibration analyses showed that the original model slightly underestimated LRR risk in that population [30].

In our validation of the INFLUENCE 3.0 model, AUCs for LRR were somewhat higher (range 0.76–0.88) and, although they also declined over time, discrimination remained good at 5 years: AUC 0.723 (95% CI 0.67–0.78) in the non-NST group and AUC 0.825 (95% CI 0.75–0.90) in the NST group. Calibration plots similarly showed overestimation of LRR risk at lower predicted probabilities.

### 4.2 Comparison of the Belgian and Dutch cohorts

The results could be related to differences in patient populations. The cohorts were similar in age, tumor sublocalisation, histology, HER2 status with targeted therapy, and chemotherapy, but differed on other factors. The Dutch cohort included patients diagnosed 2012–2016, while 65% of Leuven patients were diagnosed before 2012. Screen-detected cases were lower in Leuven (36.6% non-NST, 9.1% NST vs. 47.1% and 15.2%) and ductal carcinoma in situ (DCIS) was more frequent (73% vs. 50.7% non-NST; 50.7% vs. 35.6% NST). Leuven patients also had higher-grade tumors, more advanced stage, higher mastectomy rates, more endocrine therapy (83.5% vs. 53.6% non-NST), and more frequent axillary lymph node dissection (56.9% vs. 15.2% non-NST; 83.7% vs. 36.4% NST).

### 4.3 Clinical implications

Some miscalibration was observed, mainly overestimation of LRR and CBC risks, but absolute deviations were small due to low event rates [14,29,31]. Although this may demand for slightly intensified surveillance, the clinical impact is likely limited, and modest overestimation may be preferable to underestimation when tailoring follow-up.

In practice, discrimination is considered the more important performance aspect for INFLUENCE 3.0, as it identifies patients who may be candidates for less intensive surveillance [36]. In that regard, the model showed adequate discrimination for LRR in both NST and non-NST groups.

In contrast, CBC discrimination in the non-NST group was poor and near random, largely due to the narrow range of low predicted risks. However, because calibration was acceptable, these predictions should not be considered unreliable or misleading, they simply show limited variation. Therefore, while tailoring surveillance schemes based on CBC risk predictions is not recommended, clinicians and patients can be guided by the LRR risk estimates, which demonstrate better discrimination. This LRR risk, together with the relatively constant CBC risk, may still provide meaningful guidance in optimizing individual surveillance strategies.

### 4.4 Limitations

Some limitations are the use of historical (2000–2018), single-center data, with a relatively small sample size (particularly the NST subgroup) and few events, resulting in less stable estimates [10].

Furthermore, INFLUENCE 3.0 includes a broad set of established clinical predictors of LRR and CBC [35,[37], [38], [39], [40], [41], [42]]. However, other studies also link LRR to positive/close margins, lymphovascular invasion, and premenopausal status [43]. While these factors are not included in the model, INFLUENCE 3.0 indirectly accounts for margins and menopausal status through exclusion of patients with positive/close margins and inclusion of age, which largely reflects menopausal status.

Another limitation is incomplete information on irradical surgical margins for a subset of patients. Of approximately 300 patients with initially irradical margins, 50 were manually reviewed; only two had more-than-focally irradical margins without appropriate re-intervention and were excluded. Based on this low proportion, it was assumed that similar cases among the remaining patients were rare, and they were retained in the analysis. Nevertheless, this likely resulted in fewer exclusions than in the Dutch cohort (0.02% vs. 0.7%). As positive margins increase LRR risk, their inclusion may have led to underestimation of model performance [44,45].

Lastly, the definition of our primary outcome LRR needs some explanation. We used the same definition as the one that was used for the development of INFLUENCE, which is in line with consensus-based event definitions [18]a. However, it should be noted that in clinical practice the definitions of recurrent events vary and that there are multiple different classification systems to classify a recurrent event as a true recurrence or second primary breast cancer [46]. We chose to embrace this practical variation because treatments are likely to be based on the definition as reported in the patient files. A sensitivity analysis of our data in which we excluded the ipsilateral new primary breast cancers (which were only few events) from this definition did not alter the results.

### 4.5 Future perspectives

INFLUENCE 3.0 currently estimates 5-year recurrence risks, although LRR and CBC are known to continue occurring beyond 10 years of follow-up [47,48]. In the Leuven cohort, median follow-up was 9.8 years in the non-NST group and 6.5 years in the NST group. Over this period, 225 non-NST patients (3.0%) developed LRR and 315 (4.2%) CBC, while 39 LRRs (3.9%) and 28 CBCs (2.8%) occurred in the NST group. The number of LRR and CBC events nearly doubled with extended follow-up, underscoring the clinical relevance of extending INFLUENCE 3.0 predictions to at least 10 years in future updates.

An additional important future improvement is extending INFLUENCE 3.0 to predict distant recurrence. Although no randomized BC-specific evidence shows that earlier systemic treatment of asymptomatic metastatic disease improves outcomes—as such trials are ethically challenging—identifying patients at high risk for distant recurrence remains clinically valuable, as it reflects aggressive tumor biology. Moreover, evidence shows that local stereotactic RT in oligometastatic disease improves overall survival (OS) [49]. Earlier identification of distant recurrences could therefore support earlier symptom management preserving quality of life, and detection of oligometastatic disease, potentially improving OS [50,51]. Since INFLUENCE 2.0 already included distant recurrence prediction, integrating all recurrence types into a single, unified INFLUENCE model would be valuable to clinical decision-making and support personalised follow-up [11].

Furthermore, INFLUENCE 3.0 provides individualised LRR and CBC risk estimates, which should be interpreted by clinicians and discussed with patients to guide follow-up. However, translating absolute percentages into clinical decisions is challenging. Therefore, expanding the model to compare predicted risks with population-level benchmarks, such as the yearly LRR and CBC incidence rates summarized by Spronk et al. could allow the establishment of clinically meaningful cut-offs [47]. Patients below such thresholds might follow less intensive follow-up, while higher-risk patients could be considered for a more intensive surveillance [4,52]. Nonetheless, developing and validating such clinical guidance would require separate dedicated research, which is partly addressed by the ongoing NABOR study [53].

## 5 Conclusion

In this study, the INFLUENCE 3.0 model was externally validated using data from the University Hospitals Leuven, Belgium. While the model showed acceptable calibration and good to very good discrimination for LRR risk estimation, its discriminative ability for CBC risk estimation in the non-NST group was moderate to poor. However, because CBC risk predictions were narrowly distributed and showed acceptable calibration, the overall risk estimates from INFLUENCE 3.0 – primarily the LRR risks – could still be valuable for tailoring surveillance strategies. These findings highlight the need for further model updates such as recalibration or alternative approaches to improve CBC risk estimation, and emphasize the importance of additional external validation – ideally performed on larger datasets – to confirm model suitability across different populations and healthcare settings.

## CRediT authorship contribution statement

**Aline van der Vorst:** Writing – review & editing, Writing – original draft, Resources, Project administration. **Carlijn Grimberg:** Writing – review & editing, Writing – original draft, Investigation, Formal analysis. **Marissa C. van Maaren:** Writing – review & editing, Resources, Project administration, Data curation, Conceptualization. **Tom A. Hueting:** Writing – review & editing, Methodology. **Caroline Weltens:** Writing – review & editing, Supervision, Conceptualization. **Sabine Siesling:** Writing – review & editing, Supervision, Conceptualization. **Catharina G.M. Groothuis-Oudshoorn:** Writing – review & editing, Methodology.

## Ethics declaration

The authors have NOT obtained informed consent from participants or their legal representatives. This retrospective observational study was based on routinely collected clinical data from a registry that uses an opt-out procedure. Patients are automatically included in the registry, and their data may be used for research purposes unless they object to such use. Therefore, separate informed consent for the present study was not required.

This study was performed in compliance with relevant laws, regulatory frameworks and guidelines where the research took place. This study was approved by the BMS Ethical review committee, University of Twente, and Ethics Committee Research UZ/KU Leuven. (Approval No. 240722 (University of Twente) and S68978 (UZ Leuven))

## Data availability

Due to privacy regulations, the dataset used in this study is not publicly available. Aggregated data is available on reasonable request by contacting the corresponding author.

## Declaration of generative AI and AI-assisted technologies in the writing process

During the preparation of this work the author(s) used ChatGPT for linguistics solely. After using this tool/service, the author(s) reviewed and edited the content as needed and take(s) full responsibility for the content of the publication.

## Funding

During the preparation of this manuscript, Aline Van der Vorst received funding from the Emmanuel van der Schueren Starter Fund of 10.13039/501100011851Kom op tegen Kanker [KOTK_KUL/2024/13905]. The funder did not play any role in the design, analysis and conclusions of this study.

## Declaration of competing interest

The authors declare the following financial interests/personal relationships which may be considered as potential competing interests: Prof. Sabine Siesling is specialty editor Epidemiology at the Breast. Given her role as specialty editor, Sabine Siesling will have no involvement in the peer-review of this article and have no access to information regarding its peer review. Dr. Marissa C van Maaren, Dr. Catharina GM Groothuis-Oudshoorn and Tom A Hueting have been involved in the development of the INFLUENCE 3.0 model. If there are other authors, they declare that they have no known competing financial interests or personal relationships that could have appeared to influence the work reported in this paper.

## Footnotes

Appendix A Supplementary data to this article can be found online at https://doi.org/10.1016/j.breast.2026.104944.
