# Supplementary material for: Belgian validation of the INFLUENCE 3.0 model predicting 5-year locoregional and contralateral breast cancer recurrence
Source: Breast. 2026 Oct 7;90:104944. doi: 10.1016/j.breast.2026.104944 (PMC13675742; doi:10.1016/j.breast.2026.104944)
Supplement: Supplementary file 1 [file mmc1.docx]

## Appendix A – Missing data pattern

Missing data pattern for the non-NST group:

**Suppl. Fig. 1.** Missing data pattern in the non-NST group.

The left vertical axis shows the number of patients missing specific variables. The right vertical axis indicates how many different variables are missing. For example, 372 patients are missing one variable: the sublocalisation of the tumour. The horizontal axis shows the frequency with which each variable is missing. For example, the variable HR_stat_treat is missing in 19 cases.

Abbreviations: morf = histological tumour type, pt = pathological tumour stage, pn = pathological nodal stage, surgery_rt_reconstr = surgery type ± radiation therapy ± immediate reconstruction, chemo = chemotherapy, LRR_def = occurrence of locoregional recurrence, CBC_def = occurrence of contralateral breast cancer, HR_stat_treat = hormonal receptor status ± endocrine treatment, diffgrad = differentiation grade, her2_stat_treat = HER2 status ± targeted treatment, bvo_detec = mode of detection, sublok = tumour sublocalisation.

Of the 7,549 patients, 6,729 had no missing data, leaving 820 patients with one or more missing variables. Sublocalisation (*sublok*) and mode of detection (*bvo_detec*) were among the most frequently missing variables, with 425 and 321 missing values, respectively.

Missing data pattern for the NST group

**Suppl. Fig. 2.** Missing data pattern in the NST group.

The left vertical axis shows the number of patients missing specific variables. The right vertical axis indicates how many different variables are missing. For example, 97 patients are missing one variable: the sublocalisation of the tumour. The horizontal axis shows the frequency with which each variable is missing. For example, the variable pcr is missing in 4 cases.

Abbreviations: morf = histological tumour type, dcis_comp = presence of ductal carcinoma in situ, okd = axillary lymph node dissection, LRR_def = occurrence of locoregional recurrence, CBC_def = occurrence of contralateral breast cancer, pcr = pathologic complete response, HR_stat_treat = hormonal receptor status ± endocrine treatment, her2_stat_treat = HER2 status ± targeted treatment, diffgrad = differentiation grade, bvo_detec = mode of detection, sublok = tumour sublocalisation.

Of the 999 patients, 799 had no missing data, leaving 200 patients with one or more missing variables. Sublocalisation (*sublok*) and mode of detection (*bvo_detec*) were also in this group among the most frequently missing variables, with 119 and 74 missing values, respectively.

## Appendix B – Discrimination results for the recent (2012-2018) non-NST cohort

Due to low CBC AUCs (~0.5) in non-NST patients, additional analyses were performed. In patients diagnosed from 2012 onward, This resulted in a dataset of 2,640 patients, of whom 20 developed a CBC within 5 years. AUCs ranged 0.43–0.55.

**Suppl. Table 3.**

|  | Year 1* | Year 2 | Year 3 | Year 4 | Year 5 |
| --- | --- | --- | --- | --- | --- |
| LB | NA | 0.325 | 0.362 | 0.376 | 0.417 |
| **AUC** | **NA** | **0.490** | **0.508** | **0.510** | **0.528** |
| UB | NA | 0.654 | 0.654 | 0.644 | 0.639 |

Discrimination results for CBC-risk estimation in the recent (2012-2018) non-NST cohort.

* As there were no events in the first year, the AUC for year 1 could not be estimated.

**Suppl. Fig. 5**. Yearly AUCs for CBC-risk estimation in the recent (2012-2018) non-NST cohort. As there were no events in the first year, the AUC for year 1 could not be estimated.
